# Integrated NIRS and QTL assays reveal minor mannose and galactose as contrast lignocellulose factors for biomass enzymatic saccharification in rice

**DOI:** 10.1186/s13068-021-01987-x

**Published:** 2021-06-26

**Authors:** Zhen Hu, Youmei Wang, Jingyuan Liu, Yuqi Li, Yanting Wang, Jiangfeng Huang, Yuanhang Ai, Peng Chen, Yuqing He, Muhammad Nauman Aftab, Lingqiang Wang, Liangcai Peng

**Affiliations:** 1 Biomass and Bioenergy Research Centre, College of Plant Science and Technology, Huazhong Agricultural University, Wuhan, 430070 China; 2 Laboratory of Biomass Engineering and, Nanomaterial Application in Automobiles, College of Food Science and Chemical Engineering, Hubei University of Arts and Science, Xiangyang, China; 3 College of Resources and Environment, Huazhong Agricultural University, Wuhan, 430070 China; 4 State Key Laboratory for Conservation and Utilization of Subtropical Agro-Bioresources, College of Agriculture, Guangxi University, Nanning, 530004 China; 5 National Key Laboratory of Crop Genetic Improvement and National Centre of Plant Gene Research (Wuhan), Huazhong Agricultural University, Wuhan, 430070 China; 6 Institute of Industrial Biotechnology, GC University, Lahore, Pakistan

**Keywords:** Rice straw, Monosaccharide, Wall polymers, Biomass saccharification, NIRS modeling, QTL mapping

## Abstract

**Background:**

Identifying lignocellulose recalcitrant factors and exploring their genetic properties are essential for enhanced biomass enzymatic saccharification in bioenergy crops. Despite genetic modification of major wall polymers has been implemented for reduced recalcitrance in engineered crops, it could most cause a penalty of plant growth and biomass yield. Alternatively, it is increasingly considered to improve minor wall components, but an applicable approach is required for efficient assay of large population of biomass samples. Hence, this study collected total of 100 rice straw samples and characterized all minor wall monosaccharides and biomass enzymatic saccharification by integrating NIRS modeling and QTL profiling.

**Results:**

By performing classic chemical analyses and establishing optimal NIRS equations, this study examined four minor wall monosaccharides and major wall polymers (acid-soluble lignin/ASL, acid-insoluble lignin/AIL, three lignin monomers, crystalline cellulose), which led to largely varied hexoses yields achieved from enzymatic hydrolyses after two alkali pretreatments were conducted with large population of rice straws. Correlation analyses indicated that mannose and galactose can play a contrast role for biomass enzymatic saccharification at *P* < 0.0 l level (*n* = 100). Meanwhile, we found that the QTLs controlling mannose, galactose, lignin-related traits, and biomass saccharification were co-located. By combining NIRS assay with QTLs maps, this study further interpreted that the mannose-rich hemicellulose may assist AIL disassociation for enhanced biomass enzymatic saccharification, whereas the galactose-rich polysaccharides should be effectively extracted with ASL from the alkali pretreatment for condensed AIL association with cellulose microfibrils.

**Conclusions:**

By integrating NIRS assay with QTL profiling for large population of rice straw samples, this study has identified that the mannose content of wall polysaccharides could positively affect biomass enzymatic saccharification, while the galactose had a significantly negative impact. It has also sorted out that two minor monosaccharides could distinctively associate with lignin deposition for wall network construction. Hence, this study demonstrates an applicable approach for fast assessments of minor lignocellulose recalcitrant factors and biomass enzymatic saccharification in rice, providing a potential strategy for bioenergy crop breeding and biomass processing.

**Supplementary Information:**

The online version contains supplementary material available at 10.1186/s13068-021-01987-x.

## Background

Crop straws represent substantial lignocellulose resource convertible for biofuels and biomaterials [1]. However, lignocellulose recalcitrance basically hinders biomass enzymatic saccharification, which results in a costly lignocellulose process unacceptable for large-scale biofuel production [2]. To reduce lignocellulose recalcitrance, attempts have been undertaken by genetic modification of plant cell walls in bioenergy crops along with optimal biomass pretreatments [3, 4]. Therefore, it becomes essential to sort out the recalcitrant factors on biomass enzymatic saccharification in bioenergy crops.

Lignocellulose recalcitrance is naturally determined by plant cell wall composition, wall polymer feature, and wall network construction [5]. As the most abundant wall polymer, cellulose crystallinity has been well examined as a major factor of lignocellulose recalcitrance [6, 7]. By comparison, hemicellulose is a major wall polysaccharide, but its deposition could reduce cellulose crystallinity for enhanced biomass enzymatic saccharification under various physical and chemical pretreatments in bioenergy grasses examined [8, 9]. In particular, arabinose and xylose, two main monosaccharides of hemicellulose, could distinctively affect lignocellulose enzymatic hydrolyses, and their ratio has thus been defined as a key parameter accountable for biomass enzymatic saccharification [10, 11]. However, it has not yet been elucidated about other minor monosaccharides' roles in biomass enzymatic hydrolyses. Furthermore, despite the uronic acids of pectin have been examined with positive impact on biomass saccharification [12], it remains to explore other pectic component roles. In addition, as a major wall polymer of secondary cell walls, lignin is deposited to form lignin-carbohydrate complexes as a barrier against accession and loading of lignocellulose-degradation enzymes [13]. Hence, it is also important to explore the genetic engineering approach for improved lignin-carbohydrate complexes in bioenergy crops.

Non-cellulosic polysaccharides (hemicellulose, pectin) are mainly composed of seven neutral monosaccharides (rhamnose, fucose, arabinose, xylose, mannose, galactose, and glucose) and two hexuronic acids (glucuronic acid and galacturonic acid) [14]. Those neutral monosaccharides have been characterized to involve in the covalent cross-linking for wall network construction [15]. For instance, the feruloylated arabinose of xylan involves in cross-linking with lignin in commelinid monocots [16], whereas the mannosyl residue of glucomannan is ether-linked to lignin in softwood [16, 17]. In spinach (*Spinacia oleracea*) and beet (*Beta vulgaris*), arabinose and galactose of pectic side-chains are esterified by ferulic acid [18], which enables a cross-linking between pectin and lignin. In addition, large amounts of arabinose and galactose are found in the lignin–carbohydrate complexes of Chinese quince (*Chaenomeles sinensis*) fruit [19]. More recently, the arabinose of the non-KOH-extractable hemicellulose residues has been proposed to interact with the β-1,4-glucan chains in amorphous regions of cellulose microfibrils for reduced cellulose crystallinity in rice, wheat, *Miscanthus*, and rapeseed [6–8, 10].

Quantitative trait loci (QTL) mapping is a powerful approach for the dissection of complex traits and identification of corresponding genes in plants [20–22]. Although this approach is in principle applicable to investigate minor monosaccharides of wall polysaccharides related to lignocellulose recalcitrance and enzymatic saccharification, it has not well been conducted in bioenergy crops, mainly due to technical difficulties of determining quantitative traits related to minor monosaccharides at a large scale. Near-infrared spectroscopy (NIRS) paired with multivariate analysis offers a fast and non-invasive method to determine these traits [23]. Importantly, NIRS models have been established for determining major wall polymers of rice [24], bamboo [25], *Miscanthus* [26], and sweet sorghum [27], but it is rarely applied to assist QTL mapping for lignocellulose recalcitrant traits [28]. Thereby, it remains interesting to combine NIRS modeling with QTL mapping for investigating minor monosaccharide roles in wall network construction and lignocellulose enzymatic saccharification.

Rice is a major food crop over the world with 731 megaton straw residues per year [29]. In this study, we randomly selected a hundred of rice straws from our previously established genetic pool of a rice recombinant inbred line (RIL) population [30]. We then determined all monosaccharides contents of rice straw samples, and also detected their biomass enzymatic saccharification after two alkali pretreatments, which enabled to perform correlative analyses between monosaccharides levels and hexoses yields released from enzymatic hydrolyses. Furthermore, this study mapped QTLs for the identified lignocellulose recalcitrant factors along with the NIRS assay, and finally sorted out two minor monosaccharides as contrast factors and novel genetic loci for further genetic modification of plant cell walls in rice and bioenergy crops.

## Results

### Varied monosaccharide composition and biomass saccharification among large population of rice straws

In this study, we selected 100 rice straw samples from our previously established rice recombinant inbred line population pools [30]. Using those rice straw samples, this study initially determined all neutral monosaccharides of non-cellulosic polysaccharides by GC–MS (Table 1). As a result, all rice straw samples were of the highest xylose contents ranged from 239.74 to 290.25 μg mg^−1^, whereas they had the second high levels of arabinose from 27.50 to 39.09 μg mg^−1^ among seven monosaccharides examined, consistent with the previous findings of xylose and arabinose as two major monosaccharides of hemicellulose in grassy plants [6, 31]. By comparison, rhamnose and fucose were detected with much lower contents from 0.02 to 1.27 μg mg^−1^, which should be mainly derived from pectic polysaccharides. Among the rest of monosaccharides examined, the rice straw samples showed largely varied glucose and galactose contents about 27.03–42.49 μg mg^−1^ and 9.27–15.4 μg mg^−1^, with much lower mannose levels from 2.17 to 3.34 μg mg^−1^, consistent with the previously reported ones in rice and other grassy crops [8, 32]. It also suggested that the largest amounts of glucose should be mainly from the -1,3–1,4-glucans, which is the characteristic hemicellulose of rice straw [33]. Hence, this study demonstrated largely varied monosaccharide levels of major hemicellulose and pectin among total of 100 rice straws examined, suggesting that those rice samples should be of quite different lignocellulose recalcitrant properties.

**Table 1:** Monosaccharide content and biomass enzymatic saccharification of 100 rice straw samples

Traits	Mean ± SD^a^	Range^b^
Monosaccharides		
Rhamnose (μg mg^−1^)	0.99 ± 0.11	0.68–1.27
Fucose (μg mg^−1^)	0.99 ± 0.09	0.02–0.35
Arabinose (μg mg^−1^)	33.76 ± 2.11	27.50–39.09
Xylose (μg mg^−1^)	263.56 ± 11.19	239.74–290.25
Mannose (μg mg^−1^)	2.66 ± 0.25	2.17–3.34
Galactose (μg mg^−1^)	12.33 ± 1.44	9.27–15.4
Glucose (μg mg^−1^)	32.75 ± 3.18	27.03–42.49
Biomass enzymatic saccharification		
Glc-Rel_0.025_ (% dry matter)^c^	14.59 ± 2.20	9.92–20.92
Glc-Rel_1_ (% dry matter)^d^	27.88 ± 2.18	19.34–32.04

^a^Average ± standard deviation

^b^Minimum to maximum

^c^Glucose released after enzymatic digestion following 0.025% (m/v) NaOH pretreatment

^d^Glucose released after enzymatic digestion following 1% (m/v) NaOH pretreatment

Furthermore, this study examined biomass enzymatic saccharification by measuring hexoses (glucose) yields against dry matter (termed as Glc-Rel_0.025_ and Glc-Rel_1_) released from enzymatic hydrolysis after 0.025% and 1% NaOH pretreatments (Table 1). Based on classic chemical analyses, total of 100 rice straw samples showed the hexose yields from 9.92 to 20.92% (% dry matter) after 0.025% NaOH pretreatments, indicating a diverse biomass enzymatic saccharification of rice straws examined. Meanwhile, higher concentrations of alkali (1% NaOH) pretreatments were performed with all rice straws, and their hexoses yields were predicted from 19.34 to 32.04% (% dry matter), according to our previously established NIRS modeling [34]. The applicability of NIRS model was evaluated by checking the spectral distribution of calibration set and prediction set, and then, the predicted values of randomly selected 20 rice samples were approximate to the measured values (Additional file 1: Figure S1 A, B, C), indicating that the NIRS model should be highly applicable for predicting biomass enzymatic saccharification of rice straw samples examined in this study. As total of 100 rice straws are of largely varied monosaccharide levels and hexoses yields, those samples could be applied to further sort out lignocellulose recalcitrant impacts on biomass enzymatic saccharification.

### Distinct minor monosaccharides impacts on biomass enzymatic saccharification

**Fig. 1:**
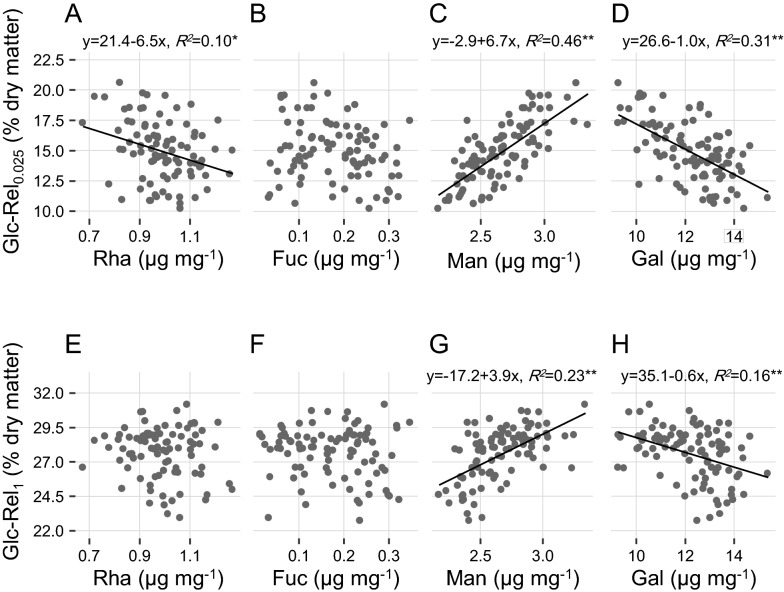
Correlation analyses between monosaccharides and glucose yields released from enzymatic hydrolyses after alkali pretreatment. **A**–**D** Correlation between rhamnose (Rha), fucose (Fuc), mannose (Man), and galactose (Gal) levels and glucose released after biomass enzymatic digestion following 0.025% (m/v) NaOH pretreatment (Glc-Rel_0.025_). **E**–**H** Correlation between Rha, Fuc, Man, and Gal levels and glucose released after biomass enzymatic digestion following 1% (m/v) NaOH pretreatment (Glc-Rel_1_). * and ** indicated significant correlations at *P* < 0.05 and 0.01, respectively (*n* = 100)

Correlation analysis has been well applied to account for wall polymer impact on biomass enzymatic saccharification in various bioenergy crops. Although the major monosaccharides (xylose, arabinose) of hemicelluloses and uronic acids of pectin have been characterized by their distinct impacts on biomass enzymatic saccharification [7, 8, 12, 35], little is yet known about minor monosaccharide roles, probably due to much less depositions into plant cell walls. However, as those 100 rice straw samples showed largely varied monosaccharides levels and biomass saccharification as described above, their correlations were performed in this study (Fig. 1). As a result, the galactose levels showed a negative correlation with the hexoses yields after 0.025% NaOH pretreatments at *P* < 0.01 level, whereas the mannose content was positively correlated with relatively higher coefficient *R*^*2*^ value among all rice straws examined (Fig. 1C, D), indicating that the mild alkali pretreatments should be suitable for sorting out the minor monosaccharide roles in biomass enzymatic hydrolyses. It also suggested that the minor monosaccharides could be effectively extracted from the alkali pretreatment at low concentration. Meanwhile, rhamnose also showed a negative correlation at *P* < 0.05 level, but its coefficient *R*^*2*^ value was extremely low, probably due to a complete extraction of rhamnose in the rice straw samples examined (Fig. 1A). Furthermore, similar correlations were observed between mannose or galactose levels and hexoses yields predicted by the NIRS assay from 1% NaOH pretreatments, but both mannose and galactose were calculated with much lower coefficient *R*^*2*^ values (Fig. 1G, H), which may be due to removal of the most mannose and galactose from 1% NaOH pretreatments. In addition, this study did not find any significant correlation between rhamnose levels and hexoses yields from the NIRS assay (Fig. 1E), and in particular, the fucose did not show any significant correlation from both chemical and NIRS assays (Fig. 1B, F), which should be mainly due to the fucose with extremely low deposition into plant cell walls. Taken together, it suggested that the galactose and mannose of wall polysaccharides could distinctively affect lignocellulose recalcitrant properties due to their contrast impacts on biomass enzymatic saccharification, whereas the rhamnose and fucose may play a small role in lignocellulose recalcitrance with little impact on biomass enzymatic hydrolysis examined.

### Potential mannose and galactose association with lignin deposition

To understand how the mannose and galactose had a contract impact on biomass enzymatic saccharification, this study also conducted a correlation analysis between two minor monosaccharides and other major wall polymers such as two types of lignin contents, three lignin monomer (H, S, G) compositions, cellulose contents, and its crystalline index (CrI) values of total 100 rice straw samples. Based on our previously established NIRS modeling [34], this study predicated acid-soluble lignin (ASL) and acid-insoluble lignin (AIL) levels after further verifying accuracy of the models (Additional file 1: Figure S1 D, E). The predicted ASL and AIL were, respectively, ranged from 1.70–3.10% to 9.36–14.73% (% dry matter), consistent with large variations of three lignin monomer levels as previously reported [36] (Additional file 2: Table S1). Meanwhile, this study also calculated largely varied cellulose contents (387.52–631.02 μg mg^−1^) and cellulose CrI values (37.61–59.33%) among total of 100 rice straw samples examined before (Additional file 2: Table S1).

**Fig. 2:**
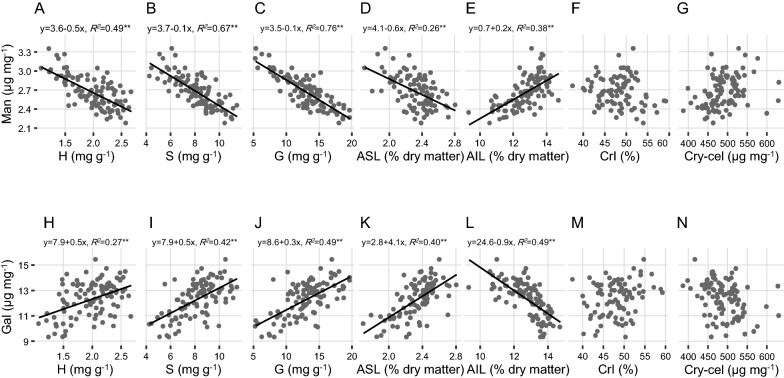
Correlation analyses of mannose (Man) and galactose (Gal) with lignin and cellulose-related traits. **A**–**G** Man correlation with ρ-hydroxy-phenyl lignin H, syringyl lignin S, guaiacyl lignin G, acid-soluble lignin (ASL), acid-insoluble lignin (AIL), cellulose crystallinity index (CrI) and crystalline cellulose (Cry-cel). **H**–**N** Gal correlation with H, S, G, ASL, AIL, CrI, and Cry-cel. * and ** indicated significant correlations at *P* < 0.05 and 0.01, respectively (*n* = 100)

According to the correlative analyses, this study calculated that the mannose levels of rice straws had either significantly negative correlations with three lignin monomers and ASL contents or positive correlations with AIL ones at *P* < 0.01 with relatively high coefficient *R*^*2*^ values (Fig. 2A–E). By contrast, the galactose levels were positively correlated with three lignin monomers and ASL contents, but had a negative correlation with AIL ones (Fig. 2H–L). The data thus suggested that the mannose should be tightly associated with AIL, whereas the galactose may be linked to ASL for wall network construction in rice straws. Furthermore, as three lignin monomers and ASL were obtained from biomass acidolysis and AIL was resistant to concentrated sulfuric acid extraction, it was understandable about either a similar correlative trend of three monomers and ASL or a contrast trend between ASL and AIL. In addition, this study found that both mannose and galactose did not show any significant correlations with cellulose levels and CrI values among rice straw samples examined (Fig. 2F, G, M, N), suggesting that these two minor monosaccharides should not directly be involved in interaction with cellulose microfibrils.

### Optimal NIRS assays for mannose and galactose at large scale

**Fig. 3:**
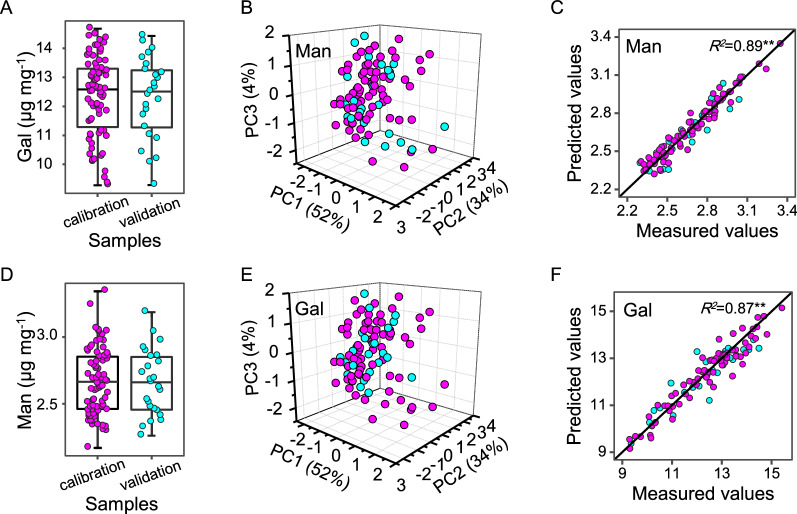
Calibration and validation of NIRS equations for predicting Man and Gal. **A**, **D** Reference values of calibration set (*n* = 75) and validation set (*n* = 25). **B**, **E** Distribution of calibration set and validation set in principal components space of spectra. **C**, **F** Correlation of predicted values and reference values of Man and Gal in calibration set and validation set. The solid line as the best linear relationship (1:1); purple circles and blue circles, respectively, as calibration set and validation set in all plots

Since minor mannose and galactose of wall polysaccharides may involve in wall–polymer interaction to distinctively affect lignocellulose recalcitrance and biomass enzymatic saccharification as described above, it becomes important to establish optimal NIRS equations for fast assay of mannose and galactose levels among large population of rice straws. First, this study divided 100 rice straw samples into calibration sets and external validation sets, based on the order of reference value. Every four ordered samples were selected and merged into the validation sets, and the remaining samples were merged into the calibration sets. Then, the calibration sets and validation sets, respectively, included 75 and 25 samples, and the reference values of two sets displayed a similar distribution (Fig. 3A, D). Besides, three-dimensional plot of spectra principal component analysis scores showed the external validation samples distributed evenly in the corresponding calibration samples (Fig. 3B, E), indicating that the calibration and external validation sets were suitable for building NIRS equations.

Furthermore, seven scatter correction methods, two derivative treatments, and three spectrum regions were combined with modified partial least-squares regression technique, leading to establishing a total of 42 calibration equations for mannose and galactose assay (Additional file 2: Table S2). The optimal equations were selected out according to the determination coefficient of calibration (*R*^*2*^*c*) with two best equations identified, which had high coefficients of calibration, cross-validation, and external validation (*R*^*2*^*c*, *R*^*2*^*cv,* and *R*^*2*^*ev*) at 0.91, 0.85, and 0.87, respectively (Table 2). The ratio performance deviation (RPD) values of these equations were also calculated at 2.50 for mannose model and 3.02 for galactose one, respectively. In addition, the scatter plots showed that the predicted and reference values of mannose and galactose were approximately equal with the best linear regression at more than 0.87 (Fig. 3C, F). Therefore, the optimal NIRS equations were acceptable for fast prediction of mannose and galactose levels among large population of rice straw samples.

**Table 2:** Calibration and validation of NIRS equations for predicting mannose and galactose of wall polysaccharides

Traits	Calibration							Cross validation			External validation		
	*N*	Spectrum (nm)	DT	SCM	Terms	*SEC*	*R*^*2*^*c*	*SECV*	*R*^*2*^*cv*	*RDP*	*N*	*SEP*	*R*^*2*^*ev*
Man	75	1108–2492	1,4,4,1	SNVD	7	0.07	0.91	0.10	0.86	2.50	25	0.10	0.87
Gal	75	1108–2492	1,4,4,1	DET	5	0.39	0.92	0.56	0.85	3.02	25	0.51	0.89

DT, derivative treatment; SCM, scatter correction methods; *SEC*, standard error of calibration; *R*^*2*^*c*, determination coefficient of calibration; *SECV*, standard error of cross-validation; *R*^*2*^*cv*, determination coefficient of cross-validation; *RPD*, ratio performance deviation; *SEP*, standard error of prediction in external validation; *R*^*2*^*ev*, determination coefficient of external validation; SNVD, a combination of standard normal variant and detrend; DET, detrend only; *N*, sample number; Terms, number of principal component used for calibration

### NIRS-assisted QTL identification for potential engineering of mannose and galactose

As both mannose and galactose could distinctively affect lignocellulose recalcitrance, it remains a challenge for their genetic modification in bioenergy rice. Using the NIRS equations established above, this study attempted to characterize the QTLs of five rice straw traits including mannose and galactose contents, ASL and AIL levels, and the hexoses yields (Glc-Rel_1_) from enzymatic hydrolyses after mild alkali pretreatments. From frequency distribution histograms, all these five traits exhibited a classic normal distribution among 215 rice lines, suggesting that the traits should be controlled by multiple genetic factors (Additional file 1: Figure S2).

**Fig. 4:**
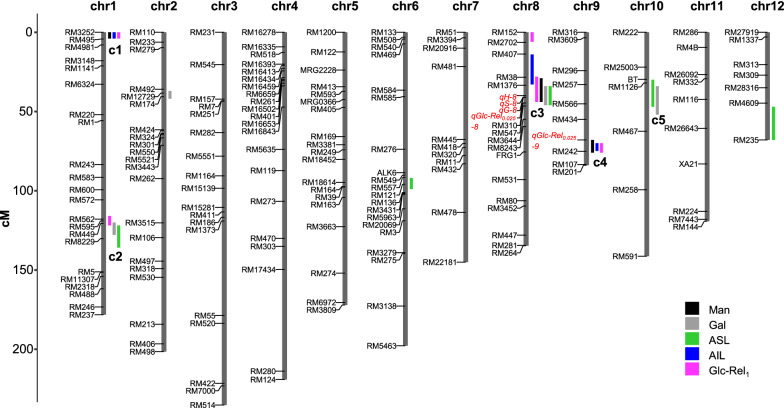
QTL mapping for cell wall factors and biomass enzymatic saccharification QTLs controlling mannose (Man), galactose (Gal), acid-soluble lignin (ASL), acid-insoluble lignin (AIL), and glucose released after biomass enzymatic digestion following 1% (m/v) NaOH pretreatment (Glc-Rel_1_) were indicated by black, gray, green, blue and purple rectangular, respectively. A genetic region which was less than 15 cM and included two or more QTLs was defined as a QTL cluster. Five QTL clusters were labeled as c1-5 in the diagram. The reported QTLs [36] are indicated in red font in the linkage map.

**Fig. 5:**
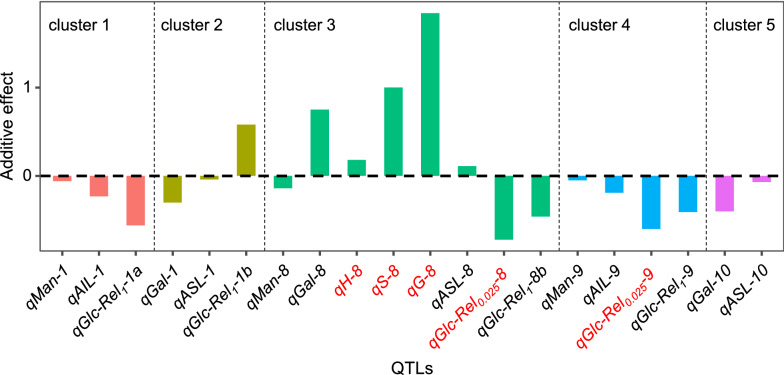
Five QTLs clusters and their additive effects. The reported QTLs were highlighted in red [36]

In general, 20 QTLs for five traits were mapped on seven chromosomes and 16 QTLs out of them were co-localized to form 5 QTL clusters (Table 3, Fig. 4). The first QTL cluster was located on the upper arm of chromosome 1 near the molecular marker RM562, and the included QTLs (*qMan-1*, *qAIL-1,* and *qGlc-Rel*_*1*_*-1a*) all displayed a negative additive effect, suggesting that the alleles from parent ZX-1 at this locus may simultaneously decrease mannose and AIL levels and hexoses yields (Glc-Rel_1_) (Fig. 5). The second QTL cluster was located on the lower arm of chromosome 1 including *qGal-1*, *qASL-1,* and *qGlc-Rel1-1b*. In comparison, the *qGlc-Rel*_*1*_*-1b* showed a positive additive effect, whereas the *qGal-1* and *qASL-1* had a negative additive effect, indicating that the alleles from parent ZX-1 at this locus should increase Glc-Rel_1_, but decrease Gal and ASL. The third QTL cluster was located on the upper arm of chromosome 8 including *qMan-8*, *qGal-8*, *qASL-8,* and *qGlc-Rel*_*1*_*-8b*. Notably, four reported QTLs controlling three lignin monomers and Glc-Rel_*0.025*_ (*qH-8*, *qS-8, qG-8,* and *qGlc-Rel*_*0.025*_*-8*) were also included in this cluster [36]. Additive effects suggest that the alleles from parent ZX-1 at this locus should decrease Man, Glc-Rel_0.025,_ and Glc-Rel_1_ but increase Gal, three lignin monomers, and ASL. The fourth QTL cluster was located on the chromosome 9 near the molecular marker RM242 including three novel QTLs (*qMan-9*, *qAIL-9*, and *qGlc-Rel*_*1*_*-9*) and a reported QTL (*qGlc-Rel*_*0.025*_*-9*) [36]. The fifth QTL cluster on the chromosome 9 included *qGal-10* and *qASL-10* (Fig. 5). QTLs in the fourth and fifth cluster all displayed negative additive effect. Significantly, the QTL co-localizations and additive effects of clustered QTLs were consistent with the correlative analyses among these traits performed above (Figs. 1 and 2, Additional file 1: Figure S3). Hence, both QTL mapping and correlation analyses have demonstrated that Man and AIL were tightly correlated for increased biomass saccharification, whereas the Gal, ASL, and lignin monomers were associated for negative impacts on biomass enzymatic hydrolysis in rice straws.

**Table 3:** Information of mapped QTLs for monosaccharides, acid-soluble/insoluble lignin, and biomass saccharification

Traits	QTL	Chr	Position (cM)	Marker interval	LOD^a^	PVE (%)^b^
Mannose	*qMan-1*	1	0–4	RM3252–RM495	3.2	5.3
	*qMan-8*	8	29–44	RM38–RM310	15.5	33.0
	*qMan-9*	9	68–76	RM434–RM107	2.1	3.4
Galactose	*qGal-1*	1	120–128	RM595–RM8229	2.5	4.6
	*qGal-2*	2	37–42	RM492–RM174	2.3	4.0
	*qGal-8*	8	34–46	RM1376–RM547	13.1	29.8
	*qGal-10*	10	34–52	RM1126–RM467	2.0	7.8
ASL	*qASL-1*	1	122–136	RM449–RM5	2.1	3.8
	*qASL-6*	6	92–99	RM557–RM136	2.2	3.9
	*qASL-8*	8	34–46	RM1376–RM547	12.2	27.3
	*qASL-10*	10	30–47	BT–RM467	3.0	8.6
	*qASL-12*	12	47–68	RM4609–RM235	2.5	6.3
AIL	*qAIL-1*	1	0–4	RM3252–RM495	2.8	5.6
	*qAIL-8*	8	14–33	RM407–RM1376	4.8	11.7
	*qAIL-9*	9	70–75	RM434–RM242	2.0	3.9
Glc-Rel_1_^c^	*qGlc-Rel*_*1*_*-1a*	1	0–4	RM3252–RM495	3.4	6.3
	*qGlc-Rel*_*1*_*-1b*	1	116–122	RM562–RM449	3.8	7.0
	*qGlc-Rel*_*1*_*-8a*	8	0–6	RM152–RM2702	4.9	10.0
	*qGlc-Rel*_*1*_*-8b*	8	28–44	RM407–RM310	2.2	4.5
	*qGlc-Rel*_*1*_*-9*	9	70–76	RM434–RM107	2.6	4.6

^a^Logarithm of odds

^b^Percentage of the trait variance explained by the QTL

^c^Glucose released after enzymatic digestion following 1% (m/v) NaOH pretreatment

## Discussion

Genetic modification of plant cell walls has been implemented to reduce lignocellulose recalcitrance in bioenergy crops [37]. As plant cell walls are of complex structures and diverse biological functions, lignocellulose modification could simply cause a penalty of plant growth and biomass yield in the most engineered crops examined [38, 39]. Hence, genetic improvement of minor wall components has been considered as a promising solution, but it requires inexpensive, high throughput, and real-time quantification methods for sorting out minor monosaccharides' impacts on biomass enzymatic saccharification. To address this issue, this study has examined large population of diverse rice straws samples, and has found out the applicable approaches for chemical and genetic analyses at large scale as well.

Although NIRS assay is well established to quickly predict major wall polymers and biomass enzymatic saccharification, it has not been applied to evaluate minor monosaccharides of wall polysaccharides in bioenergy crops. Due to diversity of 100 rice straw samples, this study could establish optimal NIRS equations for quickly predicting mannose and galactose content. Notably, with the assist of NIRS assays, this study could not only sort out mannose and galactose contrast impacts on biomass enzymatic saccharification, but it has also predicted a variety of quantitative traits in rice samples under a short-term processing. However, even though the NIRS technology is fast and robust, the applicability of NIRS model should be evaluated before being used for prediction. The applicability of NIRS model is influenced by spectral variation range of calibration set and testing set, instrument, and testing environment [40]. In this study, we kept the same instrument and method as previously described by Huang et al. [34]. The spectral distribution of testing set is included in those of calibration set for a large number of genetically modified rice samples. Meanwhile, this study verified the accuracy of predicted values by comparing the experimental and predicted values of 20 representative rice straw samples. Thereby, the NIRS approach established in this study should have a broad application for lignocellulose assay and related trait characterization in bioenergy crops.

Provided that this study has demonstrated that the correlation analysis is accountable for minor monosaccharides' impacts on biomass saccharification, it requires a further confirmation by another method. With the assist of NIRS technology, this study mapped potential QTLs controlling mannose and galactose levels, ASL and AIL contents, and hexoses yields from enzymatic hydrolyses. Significantly, QTLs for those traits were co-located, and additive effects of clustered QTLs were precisely consistent with the correlation analyses among the traits. Hence, in terms of the mannose positive impact on biomass enzymatic saccharification after alkali pretreatments, we assumed that the mannose-rich hemicellulose may assist AIL destruction and disassociation during enzymatic hydrolysis, consistent with the previous findings that mannose is involved in the galactomannan-lignin cross-linking [17]. On the other hand, the galactose-rich pectin should be easily extracted with ASL from mild alkali pretreatment, which may cause a condensed AIL association with cellulose microfibrils against cellulases enzymes accession and loading.

As the mannose and galactose of wall polysaccharides could distinctively affect biomass enzymatic saccharification, it remains interesting to improve their deposition into wall networks by exploring a powerful genetic engineering approach. Although several QTLs have been mapped for mannose and galactose, this study lacks high-density markers and annual repeats for cloning their associated genes, which should be further explored in the future.

## Conclusions

Using a total of 100 rice straws samples, this study established optimal NIRS models for quick prediction of mannose and galactose levels of wall polysaccharides. Based on correlation analysis, this work examined that the mannose levels could positively affect biomass enzymatic saccharification, whereas the galactose had a negative impact after alkali pretreatments were performed with rice straws. Integrated QTLs mapping further indicated that the mannose and galactose should distinctively associate with lignin deposition for wall network construction, interpreting how those two minor monosaccharides had a contrast role in biomass enzymatic hydrolysis. Therefore, this study has provided a powerful strategy for fast assessments of lignocellulose recalcitrant factors and biomass enzymatic saccharification at large scale in bioenergy crops.

## Methods

### Rice straw sample collection

The recombinant inbred line population with 215 F_12-13_ rice lines was developed from a cross between cultivars ‘Huahui3’ (HH-3) and ‘Zhongguoxiangdao’ (ZX) through the single seed descent method [30]. The RIL population was planted in the Huazhong Agricultural University (Wuhan, China) with a distance of 17 cm × 27 cm during the natural growing season in 2012. Straws of three individual plants for each line were collected after grain harvest. The leaves of postharvest rice straw were removed and the stems were dried at 60 °C to a constant weight. The dried stems were ground to pass through a 40-mesh screen and the powders were stored in a dry container until their use.

### Monosaccharide analysis

Monosaccharide analysis was conducted as described by Foster et al. [41]. Dried powders were washed with 70% aqueous ethanol and chloroform/methanol (1:1, v/v) solution, followed by treatment with Amylase (from Bacillus species, Sigma) and Pullulanase (from Bacillus acidopullulyticus, Sigma). The de-starched alcohol insoluble residues represented the isolated cell wall materials. Two milligrams of cell wall materials were hydrolyzed with 2 M trifluoroacetic acid (TFA) at 121 °C for 90 min. The released monosaccharides were reduced with sodium borohydride solution. The generated alditols were acetylated with acetic anhydride and pyridine at 121 °C for 20 min. Finally, the alditol acetates were analyzed by gas chromatography–mass spectrometer (GC–MS). The derivatives were separated on an SP-2380 column with a constant flow of 1.5 μg mg^−1^. Neutral monosaccharides including arabinose, fucose, galactose, glucose, mannose, rhamnose, and xylose were quantified based on standard curves created using internal monosaccharide standards.

### Biomass pretreatment and enzymatic hydrolysis

Biomass pretreatment with 0.025% (w/v) NaOH solution and subsequent enzymatic hydrolysis were conducted as previously described by Santoro et al. [42]. Dried rice material was grinded, fed, and weighed by a custom-designed robot. The powders were treated with 750 µL NaOH solution at 90 °C for 3 h in a water bath. Fifty microliters of a solution containing 0.25 µL Accellerase 1000 (Genencor, Rochester, NY) in citrate buffer (pH 4.5, 30 mM) plus 0.01% sodium azide were added for the neutralization and saccharification. Released glucose was assayed with the glucose oxidase/peroxidase (GOPOD) method (K-GLUC, Megazyme, Ireland) using 4 μL of the supernatant of the digestion reaction mixture and 64 μL of the GOPOD assay reagent.

The 1% (w/v) NaOH pretreatment and the subsequent enzymatic hydrolysis were performed as previously described by Huang et al. [34]. About 0.3 g biomass powders were incubated with 6 mL 1% NaOH (w/v) under shaking (150 rpm) at 50 °C for 2 h. The pellet was washed with water into neutral and then washed once with acetic acid–sodium acetate buffer (pH 4.8, 0.2 M). Then, the samples were hydrolyzed with 6 mL of 0.16% (w/v) mixed-cellulases (containing ≥ 6 × 10^4^ U of β-glucanase, ≥ 600 U of cellulase, and ≥ 10 × 10^4^ U of xylanase from Imperial Jade Biotechnology Co., Ltd. Ningxia 750002, China) in acetic acid–sodium acetate buffer under shaken at 150 rpm and 50 °C for 48 h. After centrifugation at 3000*g* for 5 min, the supernatants were collected for pentose and hexose assay. Hexoses were detected by the anthrone/H_2_SO_4_ method and pentoses were detected by the orcinol/HCl method. Absorbance values were detected by a UV/Vis spectrometer (Shanghai MAPADA Instruments Co., Ltd. V-1100D).

### Lignin and lignin monomer assay

Lignin was detected using a two-step acid hydrolysis method as described by Huang et al. [34]. The samples were extracted with benzene–ethanol (2:1, v/v) in a Soxhlet for 4 h, and air-dried in hood overnight. The crude cell wall samples were hydrolyzed with 67% H_2_SO_4_ (v/v) at 25 °C for 90 min under shaken at 150 rpm, and subsequently diluted to 2.88% (v/v) with distilled water and then heated at 120 °C for 60 min. The acid-soluble lignin (ASL) was measured by UV spectroscopy at 205 nm. The remaining residues were placed in a muffle furnace at 575 ± 25 °C for 4 h as acid-insoluble lignin (AIL) assay. The AIL was calculated gravimetrically as acid-insoluble residues after subtraction for ash.

Lignin monomers were analyzed using the thioacidolysis method described by Robinson and Mansfield [43]. Two milligrams of isolated cell wall material were incubated with a mixed solution containing 175 μL dioxane, 20 μL ethanethiol, and 5 μL boron trifluoride diethyl etherate for thioacidolysis. The reaction was heated at 100 °C for 4 h with gentle mixing every hour. The product was purified with water and ethyl acetate in a vortex mixer and the ethyl acetate layer was transferred. For the trimethylsilyl (TMS) derivatization, 500 μL of ethyl acetate, 20 μL of pyridine, and 100 μL of N,O-bis(trimethylsilyl) acetamide were added and the mixture was incubated for 2 h at 25 °C. The derived lignin monomers were analyzed by gas chromatography with a quadrupole mass spectrometer (Santa Clara, CA, USA). Peaks were identified by characteristic mass spectrum ions of 299 *m/*z, 269 *m*/*z*, and 239 *m*/*z* for S, G, and H monomers, respectively.

### Crystalline cellulose analysis

Crystalline cellulose content was analyzed as described by Updegraff [44]. The pellets obtained from TFA hydrolysis of biomass samples were dissolved with Updegraff reagent [acetic acid:nitric acid:water (8:1:2, v/v/v)] at 100 °C for 30 min. The washed and air-dried pellet was incubated with 67% (v/v) H_2_SO_4_ for 45 min at room temperature. The glucose of the supernatant was quantified using colorimetric anthrone assay as follows: 10 μL of supernatant, 90 μL of water, and 200 μL of freshly anthrone reagent (2 mg anthrone/mL H_2_SO_4_) were added to a 96-well polystyrene microtiter plate. For the establishment of the standard curve, standard solutions including 0 μg, 2 μg, 4 μg, 6 μg, 8 μg, and 10 μg of glucose were also added to each plate. The plate was heated at 80 °C for 30 min, and the absorption was read at 620 nm after the plate cool to room temperature. Glucose content was calculated based on the absorbance compared to the standard curve established on the same plate.

### Cellulose crystallinity index detection

Cellulose CrI was detected using X-ray diffraction method as described by Zhang et al. [9]. Powders of plant materials were laid on the glass sample holder and then analyzed by Rigaku-D/MAX instrument (Uitima III, Japan) under plateau conditions. Ni-filtered Cu Kα radiation (*λ* = 0.154056 nm) was generated at a voltage of 40 kV and a current of 40 mA, and scanned at a speed of 10°/min from 5° to 45°. The CrI was calculated according to the intensity of the 200 peak (*I*_200_, *θ* = 22.5°) and the intensity at the minimum between the 200 and 110 peaks (*I*_am_, *θ* = 18.0°).

### Calibration and validation of NIRS equation

The near-infrared reflectance spectra of RILs were collected as previously described by Huang et al. [34]. The dried samples were placed into a mini-sample cup and screened by an XDS Rapid Content™ Analyzer (FOSS, Co., LLC., Hillerod, Denmark) equipped with a dual-detector system: silicon (400–1100 nm), lead sulfide (1100–2500 nm), and the ISIscan™ software (Infrasoft International LLC., Port Mathilda, USA). The reflectance (*R*) of each sample was recorded in triplicate at wavelengths ranged from 400 to 2500 nm with 2 nm intervals. The spectral absorbance values were recorded as log1/*R*. The chemometric management of spectra data was conducted using the WinISI III software package (Version 1.50e, Infrasoft International LLC., Port Matilda, USA). A principal component analysis algorithm was carried out to identify the spectral outlier sample and to determine the structure and variability of spectral population. The global H (GH) value of each sample was determined using the measured Mahalanobis distance from mean. Samples with GH greater than 3.0 were defined as outliners and finally eliminated.

The modified partial least-squares method, two mathematical treatments, seven scatter correction methods, and three wavelength ranges were utilized for the calibration of NIRS equation. Two mathematical treatments were “0, 0, 1, 1” and “1, 4, 4, 1”, the four digits orderly represented the number of the derivative, the gap over which the derivative was calculated, the number of the first smoothing, and the number of the second smoothing. Seven methods were no scatter correction standard, standard normal variant, detrend only, combination of SNV and detrend, standard multiple scatter correction, weighted multiple scatter correction, and inverse multi scatter correction. Three wavelength ranges were 408–2492 nm, 780–2492 nm, and 1108–2492 nm. Cross-validation was conducted to select the optimal number of factors and to avoid over-fitting. In addition, one of every four samples sorted based on the laboratory value was selected for the external validation.

### QTL mapping and QTL cluster identification

Four insertion–deletion markers and 177 simple sequence repeat markers were employed to construct the genetic linkage map of the RIL population. Linkage map was constructed using the MAPMAKER/EXP version 3.0b software with linkage criterion set to logarithm of odds (LOD) threshold greater than 3.0. The genetic distances were calculated with the Kosambi map function. QTL analysis was conducted with inclusive composite interval mapping method implemented in QTL IciMapping version 4.0 based on stepwise regression [45]. Walking speed chosen for QTL detection was 1.0 cM. An LOD threshold of 2.0 was applied to detect a significant QTL. The additive effects of QTLs were estimated as half of the difference between the phenotypic values of two parents. A genetic region which was less than 15.0 cM and included two or more QTLs was defined as a QTL cluster.

### Statistical analyses

The minimum, maximum, mean, and standard deviation of all traits were calculated using the IBM SPSS Statistics 23 software (SPSS Inc., Chicago, IL, USA). The correlation analysis was performed with R software version 4.0.1. All experiments were conducted with at least three independent replicates.

## Supplementary Information

**Additional file 1: Table S1. **Cellulose and lignin related traits of 100 rice straw samples. **Table S2.** Coefficient determination of calibration of 42 mannose and galactose NIRS models

**Additional file 2: Figure S1.** Applicability test of previously established NIRS modes [35]. **A** Spectrum variation range of prediction set and modeling set. **B** Distribution of prediction set and modeling set in principal components space of spectra. **C**–**E** Accuracy verification of NIRS models by comparing experimental and predicted values of 20 rice samples. ASL, acid soluble lignin; AIL, acid insoluble lignin; Glc-Rel_1_, glucose released after biomass enzymatic digestion following 1% (m/v) NaOH pretreatment. The fitting equations and coefficients are shown at the top of the plot. * and ** as significant correlations at *P* < 0.05 and 0.01, respectively. **Figure S2.** Frequency distributions of monosaccharides, acid soluble/insoluble lignin and biomass saccharification. **A** Mannose; **B** Galactose; **C** Acid soluble lignin; **D** Acid insoluble lignin; **E** Glucose yields released from enzymatic hydrolyses after 1% NaOH pretreatments (*n* = 215). **Figure S3.** Correlation analysis between lignin/cellulose related traits and enzymatic saccharification of alkaline pretreated rice straw. (**A**–**G**) Correlations of ρ-hydroxy-phenyl lignin H, syringyl lignin S, guaiacyl lignin G, acid soluble lignin (ASL), acid insoluble lignin (AIL), cellulose crystallinity index (CrI) and crystalline cellulose (Cry-cel) with glucose released after enzymatic digestion following 0.025% (m/v) NaOH pretreatment (Glc-Rel_0.025_). (**H**–**N**) Correlations of H, S, G, ASL, AIL, CrI and Cry-cel with glucose released after enzymatic digestion following 1% (m/v) NaOH pretreatment (Glc-Rel_1_). The fitting equations and coefficients are shown at the top of the plot. * and ** indicate the correlations are significant at *p *< 0.05 and 0.01, respectively. *n* = 100

## Data Availability

All data generated or analyzed during this study are included in this published article and its additional files.

## Abbreviations

AIL: Acid-insoluble lignin
Ara: Arabinose
ASL: Acid-soluble lignin
CrI: Crystallinity index
Cry-cel: Crystalline cellulose
Fuc: Fucose
G: Guaiacyl lignin
Gal: Galactose
Glc: Glucose
Glc-Rel_0.025_: Glucose released after enzymatic digestion following 0.025% (m/v) NaOH pretreatment
Glc-Rel_1_: Glucose released after enzymatic digestion following 1% (m/v) NaOH pretreatment
H: ρ-hydroxy-phenyl lignin
LCC: Lignin-carbohydrate complexes
LOD: Logarithm of odds
MAN: Mannose
QTL: Quantitative trait loci
RIL: Recombinant inbred line
*R*^*2*^*c*: Determination coefficient of calibration
*R*^*2*^*cv*: Determination coefficients of cross-validation
*R*^*2*^*ev*: Determination coefficients of external validation
Rha: Rhamnose
RPD: Ratio performance deviation
S: Syringyl lignin
TFA: Trifluoroacetic acid
Xyl: Xylose
