# Supplementary material for: Integrated NIRS and QTL assays reveal minor mannose and galactose as contrast lignocellulose factors for biomass enzymatic saccharification in rice
Source: Biotechnol Biofuels. 2021 Jun 26;14:144. doi: 10.1186/s13068-021-01987-x (PMC8235839; doi:10.1186/s13068-021-01987-x)
Supplement: Supplementary file 1 — Additional file 1: Table S1. Cellulose and lignin related traits of 100 rice straw samples. Table S2. Coefficient determination of calibration of 42 mannose and galactose NIRS models [file 13068_2021_1987_MOESM1_ESM.docx]

Table S1 Cellulose and lignin related traits of 100 rice straw samples

| Traits ^a^ | Mean ± SD ^b^ | Range ^c^ |
| --- | --- | --- |
| H (mg g^-1^) | 1.98 ± 0.39 | 1.08 - 2.68 |
| S (mg g^-1^) | 8.06 ± 1.66 | 4.29 - 11.47 |
| G (mg g^-1^) | 13.17 ± 3.28 | 5.73 - 21.00 |
| ASL (% dry matter) | 2.37 ± 0.21 | 1.70 - 3.10 |
| AIL (% dry matter) | 13.01 ± 0.95 | 9.36 - 14.73 |
| CrI (%) | 48.22 ± 4.38 | 37.61 - 59.33 |
| Cry-cel (μg/mg) | 491.20 ± 42.68 | 387.52 - 631.02 |

^a^ H, ρ-hydroxy-phenyl lignin; S, syringyl lignin; G, guaiacyl lignin; ASL, acid soluble lignin ; AIL, acid insoluble lignin; CrI, cellulose crystallinity index; Cry-cel, crystalline cellulose;

^b^ Average ± standard deviation;

^c^ Minimum to maximum.

Table S2 Coefficient determination of calibration of 42 mannose and galactose NIRS models

| Spectrum range (nm) | Derivative treatment ^a^ | Scatter methods ^b^ | *R^2c^* ^c^ | |
| --- | --- | --- | --- | --- |
|  |  |  | Mannose | Galactose |
| 408 – 2492 | 0,0,1,1 | None | 0.69 | 0.71 |
|  | 0,0,1,1 | SNV | 0.74 | 0.75 |
|  | 0,0,1,1 | DET | 0.71 | 0.70 |
|  | 0,0,1,1 | SNVD | 0.71 | 0.74 |
|  | 0,0,1,1 | SMSC | 0.72 | 0.74 |
|  | 0,0,1,1 | WMSC | 0.74 | 0.77 |
|  | 0,0,1,1 | IMSC | 0.72 | 0.76 |
|  | 1,4,4,1 | None | 0.75 | 0.78 |
|  | 1,4,4,1 | SNV | 0.74 | 0.81 |
|  | 1,4,4,1 | DET | 0.74 | 0.84 |
|  | 1,4,4,1 | SNVD | 0.69 | 0.82 |
|  | 1,4,4,1 | SMSC | 0.74 | 0.84 |
|  | 1,4,4,1 | WMSC | 0.73 | 0.80 |
|  | 1,4,4,1 | IMSC | 0.74 | 0.84 |
| 780 - 2492 | 0,0,1,1 | None | 0.68 | 0.72 |
|  | 0,0,1,1 | SNV | 0.73 | 0.75 |
|  | 0,0,1,1 | DET | 0.70 | 0.74 |
|  | 0,0,1,1 | SNVD | 0.75 | 0.77 |
|  | 0,0,1,1 | SMSC | 0.70 | 0.73 |
|  | 0,0,1,1 | WMSC | 0.71 | 0.79 |
|  | 0,0,1,1 | IMSC | 0.70 | 0.72 |
|  | 1,4,4,1 | None | 0.78 | 0.80 |
|  | 1,4,4,1 | SNV | 0.77 | 0.81 |
|  | 1,4,4,1 | DET | 0.75 | 0.81 |
|  | 1,4,4,1 | SNVD | 0.79 | 0.81 |
|  | 1,4,4,1 | SMSC | 0.75 | 0.81 |
|  | 1,4,4,1 | WMSC | 0.72 | 0.80 |
|  | 1,4,4,1 | IMSC | 0.75 | 0.81 |
| 1108 - 2492 | 0,0,1,1 | None | 0.67 | 0.73 |
|  | 0,0,1,1 | SNV | 0.73 | 0.76 |
|  | 0,0,1,1 | DET | 0.72 | 0.79 |
|  | 0,0,1,1 | SNVD | 0.71 | 0.78 |
|  | 0,0,1,1 | SMSC | 0.73 | 0.78 |
|  | 0,0,1,1 | WMSC | 0.70 | 0.78 |
|  | 0,0,1,1 | IMSC | 0.73 | 0.76 |
|  | 1,4,4,1 | None | 0.82 | 0.77 |
|  | 1,4,4,1 | SNV | 0.83 | 0.80 |
|  | 1,4,4,1 | DET | 0.82 | 0.85 |
|  | 1,4,4,1 | SNVD | 0.86 | 0.84 |
|  | 1,4,4,1 | SMSC | 0.82 | 0.84 |
|  | 1,4,4,1 | WMSC | 0.72 | 0.81 |
|  | 1,4,4,1 | IMSC | 0.82 | 0.84 |

^a^ The four digits orderly represent the number of the derivative, the gap over which the derivative was calculated, the number of the first smoothing, and the number of the second smoothing;

^b^ None, no scatter correction standard; SNV, standard normal variant; DET, detrend only; SNVD, combination of SNV and detrend; SMSC, standard multiple scatter correction; WMSC, weighted multiple scatter correction; IMSC, inverse multiple scatter correction;

^c^ Determination coefficient of calibration.
