# Supplementary material for: Integrated NIRS and QTL assays reveal minor mannose and galactose as contrast lignocellulose factors for biomass enzymatic saccharification in rice
Source: Biotechnol Biofuels. 2021 Jun 26;14:144. doi: 10.1186/s13068-021-01987-x (PMC8235839; doi:10.1186/s13068-021-01987-x)
Supplement: Supplementary file 2 — Additional file 2: Figure S1. Applicability test of previously established NIRS modes [35]. A Spectrum variation range of prediction set and modeling set. B Distribution of prediction set and modeling set in principal components space of spectra. C–E Accuracy verification of NIRS models by comparing experimental and predicted values of 20 rice samples. ASL, acid soluble lignin; AIL, acid insoluble lignin; Glc-Rel1, glucose released after biomass enzymatic digestion following 1% (m/v) NaOH pretreatment. The fitting equations and coefficients are shown at the top of the plot. * and ** as significant correlations at P < 0.05 and 0.01, respectively. Figure S2. Frequency distributions of monosaccharides, acid soluble/insoluble lignin and biomass saccharification. A Mannose; B Galactose; C Acid soluble lignin; D Acid insoluble lignin; E Glucose yields released from enzymatic hydrolyses after 1% NaOH pretreatments (n = 215). Figure S3. Correlation analysis between lignin/cellulose related traits and enzymatic saccharification of alkaline pretreated rice straw. (A–G) Correlations of ρ-hydroxy-phenyl lignin H, syringyl lignin S, guaiacyl lignin G, acid soluble lignin (ASL), acid insoluble lignin (AIL), cellulose crystallinity index (CrI) and crystalline cellulose (Cry-cel) with glucose released after enzymatic digestion following 0.025% (m/v) NaOH pretreatment (Glc-Rel0.025). (H–N) Correlations of H, S, G, ASL, AIL, CrI and Cry-cel with glucose released after enzymatic digestion following 1% (m/v) NaOH pretreatment (Glc-Rel1). The fitting equations and coefficients are shown at the top of the plot. * and ** indicate the correlations are significant at p < 0.05 and 0.01, respectively. n = 100 [file 13068_2021_1987_MOESM2_ESM.pptx]

## Slide 1
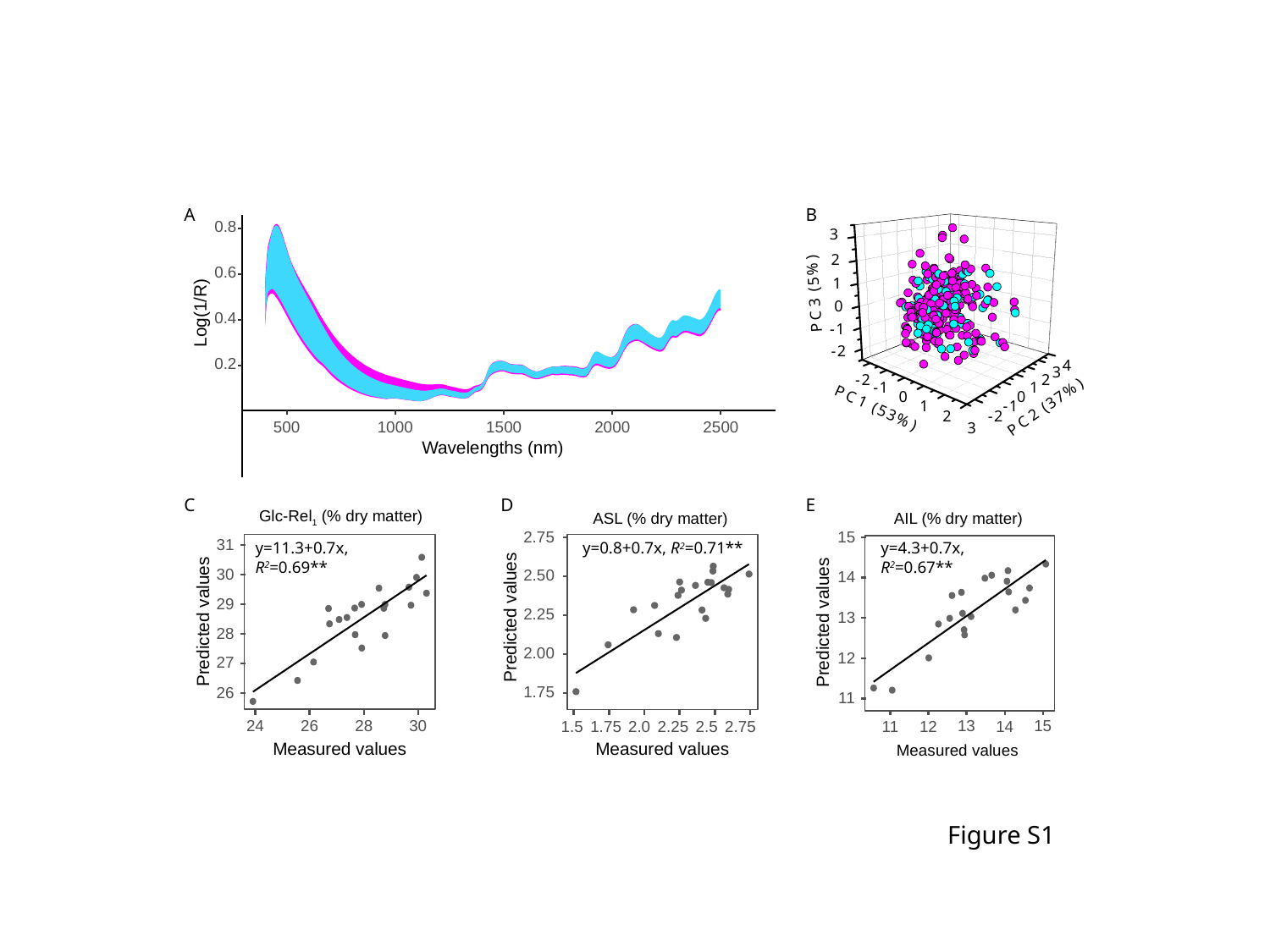

A
B
0.8
0.6
Log(1/R)
0.4
0.2
500
1000
1500
2000
2500
Wavelengths (nm)
C
D
E
Glc-Rel1 (% dry matter)
y=11.3+0.7x, R2=0.69**
31
30
29
Predicted values
28
27
26
30
26
28
24
Measured values
ASL (% dry matter)
2.75
y=0.8+0.7x, R2=0.71**
2.50
2.25
Predicted values
2.00
1.75
1.5
1.75
2.0
2.25
2.5
2.75
Measured values
AIL (% dry matter)
15
y=4.3+0.7x, R2=0.67**
14
13
Predicted values
12
11
13
15
11
12
14
Measured values
Figure S1

## Slide 2
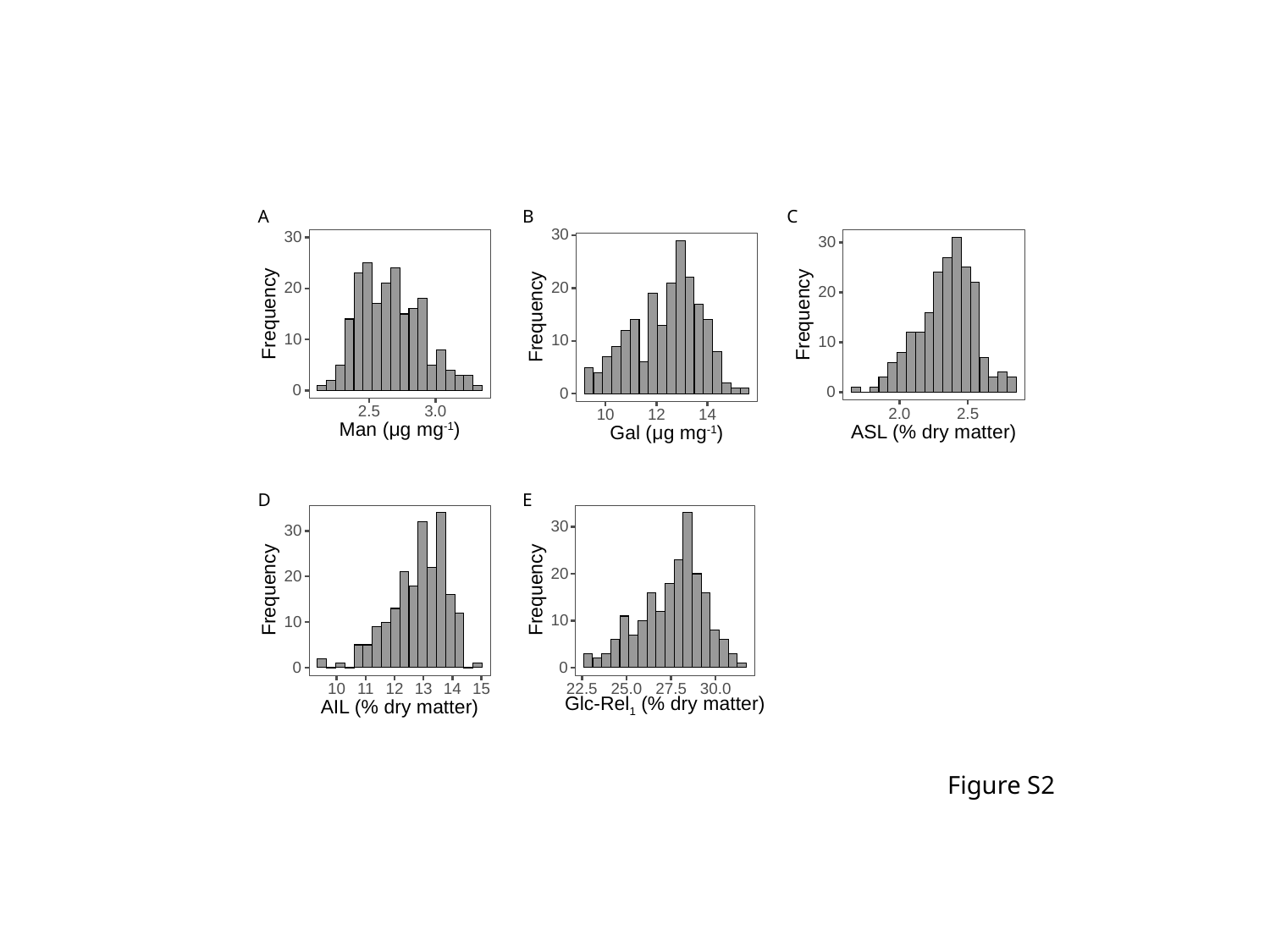

A
B
C
30
20
Frequency
10
0
3.0
2.5
Man (μg mg-1)
30
20
Frequency
10
0
10
12
14
Gal (μg mg-1)
30
20
Frequency
10
0
2.0
2.5
ASL (% dry matter)
D
E
30
20
Frequency
10
0
13
10
15
11
12
14
AIL (% dry matter)
30
20
Frequency
10
0
30.0
22.5
25.0
27.5
Glc-Rel1 (% dry matter)
Figure S2

## Slide 3
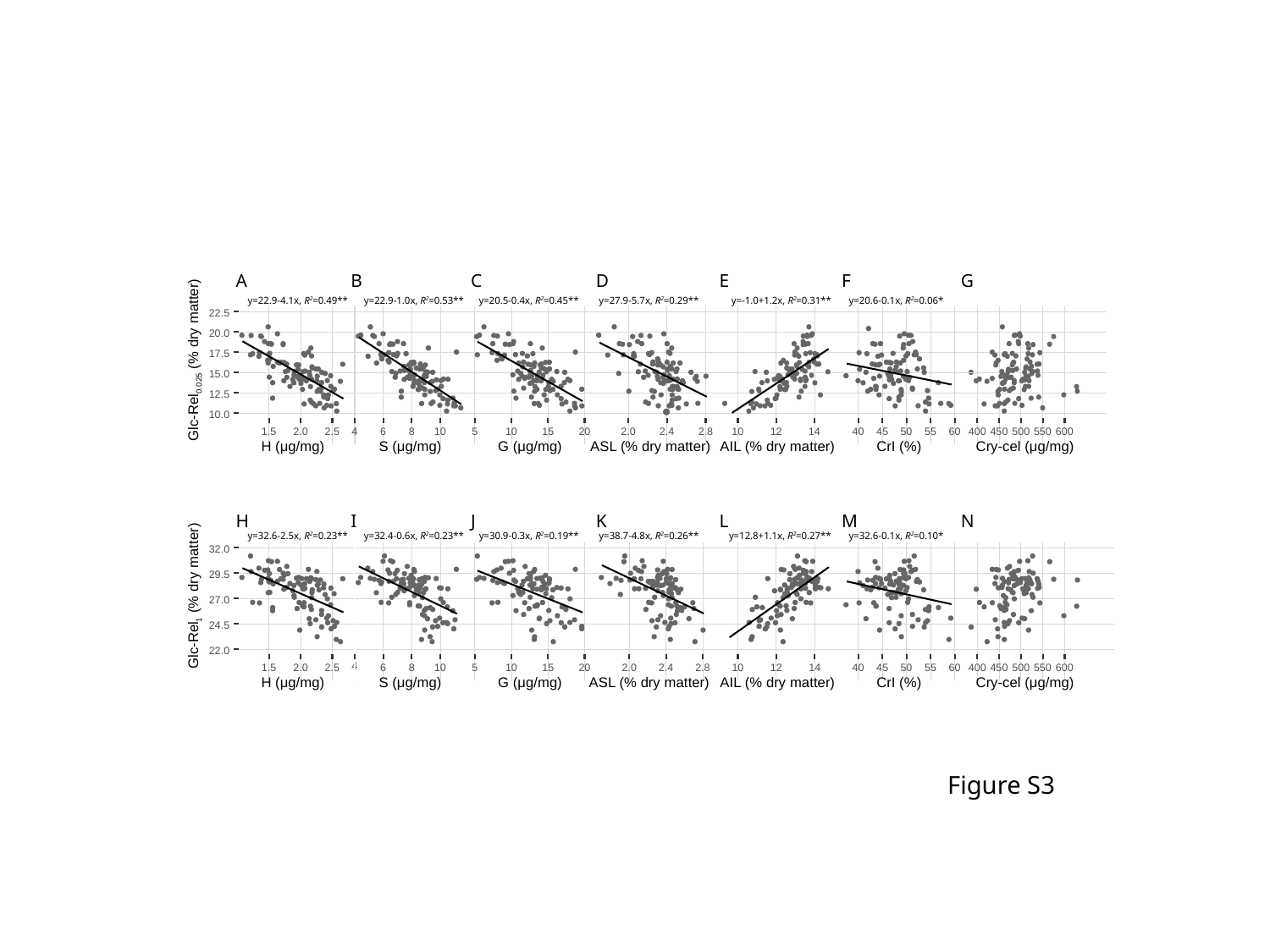

A
B
C
D
E
F
G
22.5
20.0
17.5
Glc-Rel0.025 (% dry matter)
15.0
12.5
10.0
1.5
2.0
2.5
H (μg/mg)
y=22.9-4.1x, R2=0.49**
y=22.9-1.0x, R2=0.53**
6
8
10
4
S (μg/mg)
y=20.5-0.4x, R2=0.45**
10
15
20
5
G (μg/mg)
y=27.9-5.7x, R2=0.29**
2.0
2.8
2.4
ASL (% dry matter)
y=-1.0+1.2x, R2=0.31**
10
12
14
AIL (% dry matter)
y=20.6-0.1x, R2=0.06*
40
50
60
45
55
CrI (%)
400
450
500
550
600
Cry-cel (μg/mg)
H
I
J
K
L
M
N
32.0
29.5
Glc-Rel1 (% dry matter)
27.0
24.5
22.0
1.5
2.0
2.5
H (μg/mg)
y=32.6-2.5x, R2=0.23**
y=32.4-0.6x, R2=0.23**
4
6
8
10
S (μg/mg)
y=30.9-0.3x, R2=0.19**
10
15
20
5
G (μg/mg)
y=38.7-4.8x, R2=0.26**
2.0
2.8
2.4
ASL (% dry matter)
y=12.8+1.1x, R2=0.27**
10
12
14
AIL (% dry matter)
y=32.6-0.1x, R2=0.10*
40
50
60
45
55
CrI (%)
400
450
500
550
600
Cry-cel (μg/mg)
Figure S3
